# Host-directed approaches in the pursuit of a cure for HIV

**DOI:** 10.1016/j.antiviral.2025.106216

**Published:** 2025-06-20

**Authors:** Rory A. Shepherd, Kiho Tanaka, Hannah A.D. King, Maya D. Schou, Oscar H. Lloyd Williams, Youry Kim, Michael Roche, Sharon R. Lewin

**Affiliations:** a Department of Infectious Diseases, The University of Melbourne at the Peter Doherty Institute for Infection and Immunity, Melbourne, Australia; b Department of Infectious Disease, Aarhus University, Aarhus, Denmark; c Infectious and Inflammatory Diseases Theme, School of Health and Biomedical Sciences, RMIT University, Melbourne, Australia; d Victorian Infectious Diseases Service, Royal Melbourne Hospital at the Peter Doherty Institute for Infection and Immunity, Melbourne, Australia; e Department of Infectious Diseases, Alfred Hospital and Monash University, Melbourne, Australia

## Abstract

The success of antiretroviral therapy (ART) for people with HIV has been a result of direct acting antiviral small molecules that target key components of the viral life cycle, however ART must be taken life long and there is no cure. The major barrier to a cure for HIV is the persistence of a long lived and proliferating reservoir of latently infected cells that persist on ART. Cure strategies for HIV currently target host proteins to either reduce the size of the reservoir or enhance HIV-specific immunity. A major challenge of targeting a host protein is the lack of specificity for HIV and therefore increased risk of adverse events. However, cure strategies are designed to be time limited, as opposed to ART which is lifelong. Here we review host-directed cure strategies that modulate HIV transcription and infection, enhance cell death and/or increase HIV-specific immune control. Ultimately a cure strategy will require a combination of these interventions.

## 1. Approaches to HIV cure

The advent of antiretroviral therapy (ART) for the treatment of Human Immunodeficiency Virus (HIV) infection has led to marked improvements in morbidity and mortality for people living with HIV (PLWH) such that life expectancy of PLWH is approaching that of people without HIV (Antiretroviral Therapy Cohort, 2017; Trickey et al., 2024). The success of ART has come largely through direct acting antiviral small molecules that target key components of the viral life cycle, including inhibitors of reverse transcriptase, protease, integrase and fusion; and more recently, an inhibitor of the viral capsid protein (Link et al., 2020). Targeting host factors can also successfully inhibit HIV replication as demonstrated by inhibiting viral attachment to one of the chemokine co-receptors utilised by HIV, CCR5, using the CCR5 antagonist maraviroc (Dorr et al., 2005). However, HIV infection cannot be cured by ART due to the presence of a pool of ‘latently’ infected cells where the viral genome is integrated into the host chromosome, but not actively producing viral particles. This latent reservoir is remarkably stable and may be capable of persisting indefinitely resulting in rapid rebound of virus when ART is stopped (de Jong et al., 1997; Finzi et al., 1997; Chun et al., 1999a).

**Fig. 1. –:**
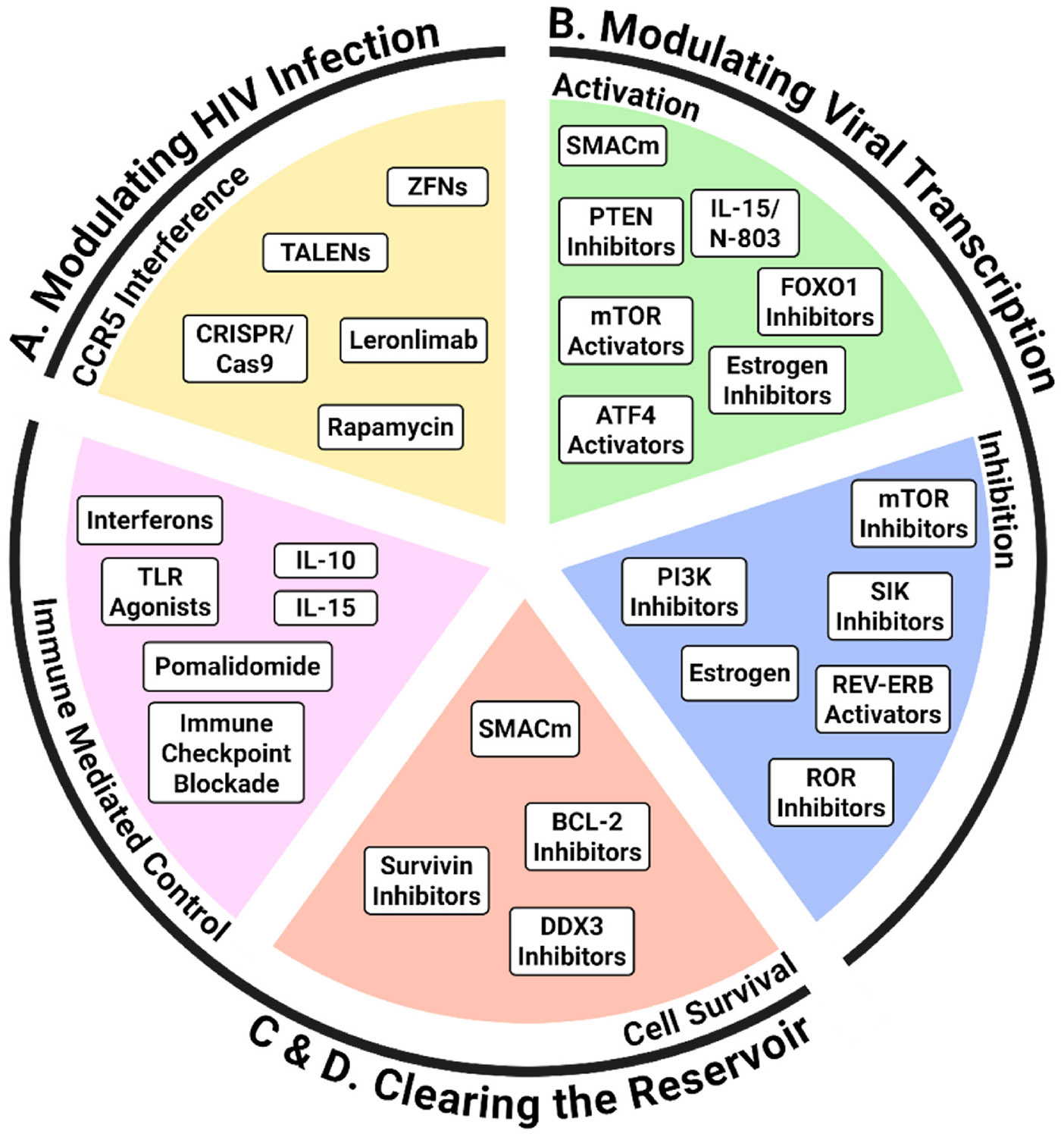
Schematic of host factors targeted in HIV cure research. Three main strategies are described in the outer circle which include A Modulation of HIV infection; B Modulation of HIV transcription; C&D Clearing the reservoir. The inner circle reflects the approaches in each strategy which include CCR5 interference; Activation; Inhibition; Cell survival; and Immune mediated control. The classes of compounds for each approach that are being investigated are listed inside the coloured segments of the circle. Forkhead box O1 (FOXO1); activating transcription factor 4 (ATF4); Mimetics of the second mitochondrial derived activator of caspases (SMACm); mammalian target of rapamycin (mTOR); phosphatase and tensin homolog (PTEN), Interleukin 15 (IL-15); retinoic acid receptor-related orphan receptors (RORs); phosphoinositide 3-kinase (PI3K); salt inducible kinase (SIK); zinc finger nuclease (ZFN); transcription activator-like effector nucleases (TALENs); clustered regularly interspaced short palindromic repeat (CRISPR) and CRISPR associated protein 9 (Cas9); DEAD-box polypeptide 3 (DDX3); B cell lymphoma 2 (BCL-2); toll like receptor (TLR) agonists; interleukin 10 (IL-10); interleukin 15 (IL-15); immune checkpoint blockade (ICB). Created in BioRender. Shepherd, R. (2025) https://BioRender.com/u33e763.

A range of approaches to HIV cure have been proposed, which can be divided into two types; those that seek to reduce the number of infected cells that contain rebound-competent virus and those that seek to enhance anti-HIV immunity which can either eliminate the HIV reservoir or rapidly respond to any emerging virus. Many of these approaches target viral proteins which include broadly neutralizing antibodies (reviewed in (Frattari et al., 2023)) and chimeric antigen receptor (CAR) T cells (reviewed in (Campos-Gonzalez et al., 2023)) that target the viral envelope protein; therapeutic vaccines (Chen and Julg, 2020) that target viral proteins through induction of HIV-specific T cells or antibodies; targeted activator of cell kill (TACK) molecules (Balibar et al., 2023); and gene-editing based strategies aimed at the integrated provirus (reviewed in (Hussein et al., 2023)). Targeting host factors can also be exploited to eliminate infected cells and boost HIV immunity to achieve an HIV cure and will be the focus of this review. Host factor targeting can modulate HIV infection; activate or inhibit HIV transcription; and clear the HIV reservoir either through targeting cell survival pathways or enhancing immune mediated control (Fig. 1).

## 2. Host targets that modulate HIV infection

### 2.1. CCR5

**Fig. 2. –:**
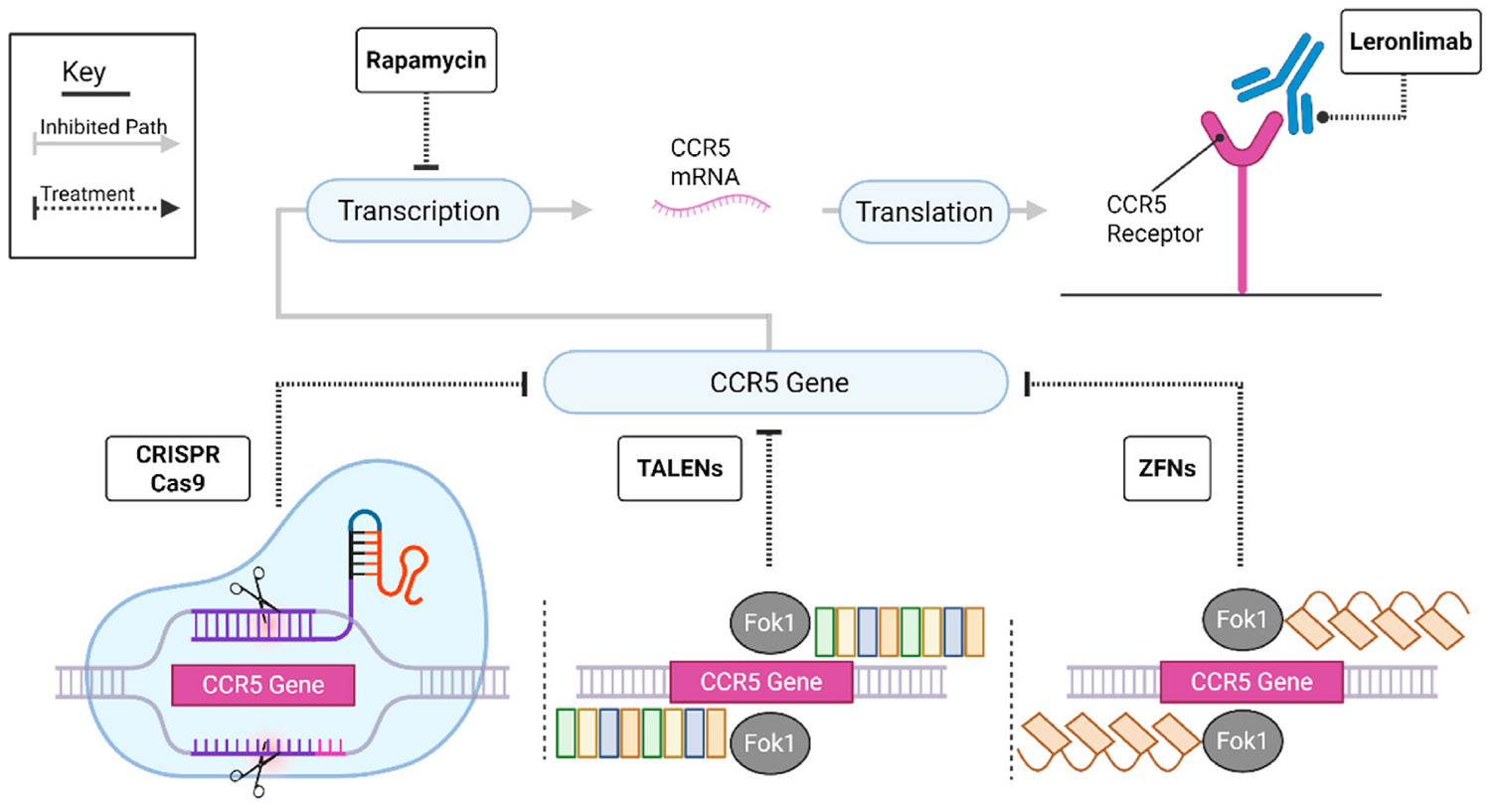
Schematic of strategies to interfere with CCR5 expression. CCR5 gene-editing has been performed using methods including clustered regularly interspaced short palindromic repeats (CRISPR)/CRISPR associated protein 9 (Cas9) modification, transcription activator effector like nucleases (TALENs) composed of 19 DNA binding domains complexed with a Fok1 endonuclease, and zinc finger nucleases (ZFNs) composed of four zinc finger proteins with a Fok1 endonuclease. Rapamycin downregulates CCR5 mRNA expression and subsequent surface expression. Leronlimab binds CCR5 and occludes HIV virion binding. Created in BioRender. Shepherd, R. (2025) https://BioRender.com/jqe8tll.

Given the successful cure of seven people with HIV following stem cell transplantation from donors who carry the Δ32 mutation in the CCR5 gene (reviewed in (Maurya et al., 2024)), there has been growing interest in reducing or eliminating CCR5 expression on CD4^+^ T cells using gene editing strategies (Fig. 2). The rationale is to reduce the pool of CD4^+^ T cells susceptible to HIV infection once ART is stopped. Various gene editing approaches have been used including: transcription activator-like effector nucleases (TALENs (Mock et al., 2015; Shi et al., 2017; Romito et al., 2021; Schwarze et al., 2021),); zinc finger nucleases (ZFNs (Tebas et al., 2014; 2021),); and clustered regularly interspaced short palindromic repeats (CRISPR)/CRISPR associated protein 9 (Cas9) (Ran et al., 2013; Xu et al., 2017, 2019).

The first phase I clinical trial of ZFN-mediated CCR5 *ex vivo* gene editing, followed by autologous reinfusion, resulted in 11–28 % of CD4^+^ T cells being CCR5 modified (Tebas et al., 2014). In a subsequent similar trial that included ART interruption, viral rebound in the participants was significantly delayed compared to historical controls (Tebas et al., 2021). To date, CRISPR/Cas9 for CCR5 has not yet been evaluated in clinical trials, but in HIV-infected humanised mice there was a 2.5 log reduction in HIV RNA after receipt of CCR5 modified CD4^+^ T cells (Xu et al., 2017). In a single recipient who received CRISPR/Cas9 CCR5 modified cells as part of treatment for acute lymphocytic leukemia the modified cells were long lived (19 months), but only 5 % of circulating lymphocytes were successfully modified (Xu et al., 2019). A recent study aimed to broaden the protection of this therapy to CXCR4 tropic viruses by combining CCR5 CRISPR/Cas9 with the C46 HIV fusion inhibitor, which resulted in greater protection against infection (Khamaikawin et al., 2024).

Suppression of CCR5 mRNA is another modality by which CCR5 expression has been reduced. Treatment with rapamycin, inhibiting its target mTOR (mammalian target of rapamycin), reduced CCR5 surface expression in CD4^+^ T cells *in vitro* (Heredia et al., 2003), in humanised mice (Heredia et al., 2015) and in cynomolgus macaques (Gilliam et al., 2007). In a humanised mouse model of acute HIV infection, rapamycin reduced CCR5 expression leading to reduced loss of CD4^+^ T cells and a smaller HIV reservoir (Nicoletti et al., 2009). Rapamycin also inhibited CD4^+^ T cell proliferation, as seen in ART-treated simian immunodeficiency virus (SIV) infected rhesus macaques, but this did not result in reduction of the viral reservoir (Varco-Merth et al., 2022).

A more broadly applicable strategy to reduce viral infection has been to target the host protein CCR5 with antibodies that occlude the viral binding site. Leronlimab (previously known as PRO140), tested in a phase II clinical study suppressed viral replication in the absence of ART in 23/41 participants over 12 weeks with no sign of anti-drug antibody development (Dhody et al., 2018). Leronlimab fully occupied surface CCR5 in both human (Chang et al., 2021b, 2022) and rhesus macaque studies (Chang et al., 2021a, 2021b, 2022) conferring protection from continued viral replication. Leronlimab has recently been delivered as a gene therapy via adeno-associated virus in an infant macaque model and demonstrated prolonged replication inhibition of the hybrid virus of SIV and HIV (SHIV) in two of the four rhesus macaques tested (Webb et al., 2024). The strategies discussed here are summarised in Table 1 with relevant small molecule compound structures displayed in Supplementary Fig. 1A.

## 3. Host factors that modulate HIV transcription

**Fig. 3. –:**
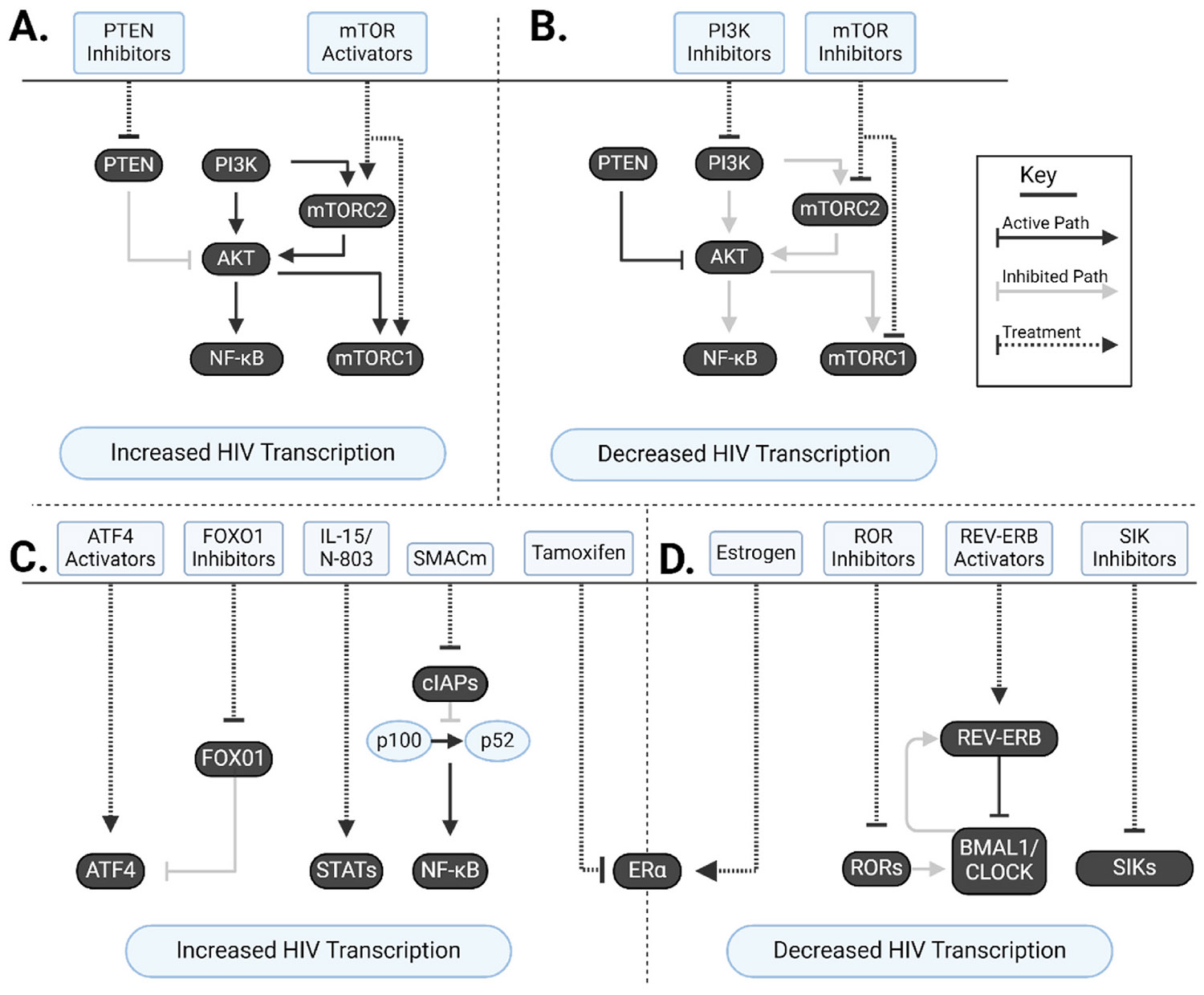
Host pathways targeted to modulate viral transcription. (A) Schematic of enhanced HIV transcription induced by PI3K/AKT/mTOR (PAM) signalling. Inhibition of phosphatase and tensin homolog (PTEN) relieves PTEN mediated suppression of the phosphoinositide 3-kinase (PI3K) and protein kinase B (AKT) signalling path, facilitating viral reactivation via NF-κB. Mammalian target of rapamycin (mTOR) activators target mTOR protein complexes 1 and 2 (mTORC1/2), inducing mTORC2 mediated transcription, as well as mTORC1 mediated activation of AKT, which in turn activates NF-κB mediated transcription. (B) Schematic of inhibited HIV transcription induced by PAM inhibitors. PI3K inhibition reduces downstream AKT and mTORC1 signalling, reducing overall NF-κB mediated transcription. mTOR inhibition similarly reduces NF-κB mediated transcription downstream of mTORC2, as well as inhibiting mTORC1 mediated transcription. (C) Schematic of other Latency Reversing Agent (LRA) induced viral transcription. Activating transcription factor 4 (ATF4) mediated activation can be induced through either direct ATF4 activators, or indirectly through inhibition of FOX01, removing its inhibition of ATF4. Signalling of interleukin 15 (IL-15) or the IL-15 superagonist N-803 triggers signal transducer and activator of transcription (STAT) mediated transcription. Mimetics of the second mitochondrial derived activator of caspases (SMACm) induce the degradation of inhibitor of apoptosis proteins (IAPS), leading to a signal cascade where RelB dimerises with p100 and drives NF-κB mediated transcription. The estrogen receptor alpha (ERα) modulator Tamoxifen reduces ERα mediated repression, thus driving an increase in transcription. (D) Schematic of other Latency Promoting Agent (LPA) repression of viral transcription. Estrogen signalling agonists increase ERα mediated repression of transcription. Inhibition of retinoic acid receptor-related orphan receptors (RORs) leads to a reduction in both ROR mediated, but also BMAL1/CLOCK mediated transcription. Activation of negative circadian factor REV-ERB inhibits BMAL1/CLOCK mediated viral transcription. Inhibitors of salt inducible kinases (SIKs), which are negative regulators of circadian signalling, reduce viral transcription. Created in BioRender. Shepherd, R. (2025) https://BioRender.com/tcpqj4i.

One major approach being investigated in HIV cure is to either enhance viral transcription from a silent provirus (often called “Shock and Kill”) or to permanently silence transcription (often called “Block and Lock”). The goal of activating the latent provirus, with latency reversing agents (LRAs), is to eliminate the infected cell either through immune mediated clearance or viral cytotoxicity (reviewed in (Zerbato et al., 2019)). Alternatively, silencing the HIV proviruses, using latency promoting agents (LPAs), aims to achieve permanent latency so that virus is unable to rebound once ART is stopped (reviewed in (Vansant et al., 2020)). HIV transcription from an integrated provirus utilises both viral factors (transactivator of transcription (Tat) and Rev proteins) as well as host machinery for transcription initiation, elongation, splicing and translation; thus, providing an opportunity to modulate these host factors allows for the activation or inhibition of HIV transcription. In relation to host factors that modulate HIV transcription, we will focus on host factors (Fig. 3) other than epigenetic regulators which impact the HIV promoter, the long terminal repeat (LTR), such as histone acetylation and methylation that have recently been reviewed elsewhere (see (Mbonye and Karn, 2024)).

### 3.1. Non-canonical NF-κB signalling

Mimetics of the second mitochondrial derived activator of caspases (SMACm, Supplementary Fig. 1B) can reactivate latent HIV as well as activate cell death pathways and are therefore of interest in HIV cure strategies. SMACm compounds activate the non-canonical NF-κB signalling pathway by inducing the degradation of baculoviral inhibitor of apoptosis (IAP) repeat containing protein 2 (BIRC2), leading to the degradation of p100 to p52, which when dimerised with NF-κB subunit RelB translocates into the nucleus and drives transcription from the viral LTR (reviewed in (Wong and Jiang, 2021), Fig. 3C). Monovalent SMACm bind the baculoviral IAP repeat 3 (BIR3) domain of X-linked IAP (XIAP) and cellular IAPs (cIAPs) (Zobel et al., 2006) while bivalent SMACm contain a linker between two binding motifs and demonstrate around 100–300 fold more potent inhibition of IAPs compared to monovalent SMACm, as well as extending interactions to BIR2 in addition to BIR3 domains (Sun et al., 2007; Lu et al., 2008).

Mono and bivalent SMACm can reverse latency in latently infected cell lines (Pache et al., 2015, 2020; Nixon et al., 2020), but have lower potency in peripheral blood mononuclear cells (PBMCs) from PLWH on ART examined *ex vivo* (Campbell et al., 2018, 2020; Nixon et al., 2020; Falcinelli et al., 2022). The reasons why SMACm have high potency in cell lines and low potency *ex vivo* in primary cells is not currently understood. However, *in vivo*, the bivalent SMACm AZD5582 induced significant blips in HIV RNA in the plasma and in the tissue of ART-treated HIV-infected humanised mice and ART-treated SIV-infected rhesus macaques (Nixon et al., 2020; Dashti et al., 2023). Interestingly, AZD5582 did not induce viral blips in SIV-infected rhesus macaques (Dashti et al., 2020). HIV RNA blips in plasma were also observed in ART-treated HIV-infected humanised mice following the administration of another bivalent SMACm, APG-1387 (Jain et al., 2024). Similarly, the monovalent SMACm xevinapant/Debio1143 also induced HIV RNA blips in a humanised mouse model and also increased supernatant HIV RNA production, comparable to anti-CD3/CD28 stimulation, in CD4^+^ T cells derived from PLWH on ART (Bobardt et al., 2019). Taken together there is evidence that SMACm can reverse latency with variable potency in latently infected cell lines, primary cells from PLWH on ART and *in vivo* in ART-treated HIV-infected mice and non-human primates.

To enhance depletion of the HIV reservoir, the bivalent SMACm AZD5582 was combined with FH1321, an inhibitor of the DEAD-box polypeptide 3 (DDX3). DDX3 is an RNA dependent helicase, and regulator of apoptosis (covered below). Co-treatment of AZD5583 with FH1321 reduced the number of infected cells that can produce infectious virus in *ex vivo* CD4^+^ T cells from PLWH (Jansen et al., 2024). In ART-treated SIV-infected rhesus macaques, AZD5582, together with SIV Env specific antibodies and an interleukin 15 (IL-15) superagonist (N-803), was able to reduce SIV DNA levels in CD4^+^ T cells (Dashti et al., 2023); however, the investigators did not stop ART so it was unclear if this intervention ultimately led to a delay in viral rebound or a change in viral set point.

One barrier to the use of bivalent SMACm in clinical trials is the challenge of dose limiting toxicities (reviewed in (Fulda, 2015)). The bivalent SMACm, birinapant, resulted in adverse neurological events, including Bell’s palsy in several clinical trials ((Amaravadi et al., 2015; Noonan et al., 2016),NCT02288208), whilst the combination of birinapant with pembrolizumab was terminated after futility analysis (NCT02587962). In contrast to clinical trials of bivalent SMACm, several clinical trials have demonstrated an acceptable safety profile of monovalent SMACm including successful phase I studies of the monovalent SMACm GDC0152 (Flygare et al., 2012); GDC0917 (Tolcher et al., 2016) (NCT01226277); LCL161 (Infante et al., 2014; Pemmaraju et al., 2021); and xevinapant/debio1143 (Hurwitz et al., 2015; Sun et al., 2020; Tao et al., 2023)). However, due to a lack of anti-tumour effects, many monovalent SMACm are now being investigated for cancer treatments in combination with other agents (reviewed in (Morrish et al., 2020)). For example, anti-PD1 antibodies are being investigated in combination with the SMACm; xevinapant (NCT03871959, NCT04122625); and LCL161 (NCT03111992, NCT02890069).

### 3.2. PAM pathway

The PI3K/AKT/mTOR (PAM) axis is a highly conserved signal transduction network in higher eukaryotic cells which modulates cell survival growth, and proliferation (reviewed in (Glaviano et al., 2023)). The pathway is composed of three major proteins including phosphoinositide 3-kinase (PI3K), protein kinase B (AK strain transforming; AKT) and mTOR (reviewed in (Diehl and Schaal, 2013; Pasquereau and Her-bein, 2022)). Each of these pathways can regulate HIV transcription and are therefore relevant for cure strategies (see Fig. 3A and B).

#### 3.2.1. Phosphatase and tensin homolog (PTEN)

PTEN is a negative regulator of the PI3K/AKT pathway and inhibits tumour necrosis factor (TNF) induced NF-κB transcription (Mayo et al., 2002). HIV replication actively downregulates PTEN (Chugh et al., 2008), reducing PTEN mediated suppression which therefore promotes viral transcription. The drug disulfiram inhibits PTEN, which enhanced NF-κB mediated activation of HIV transcription *in vitro* (Doyon et al., 2013) and in two clinical trials enrolling PLWH on ART (Elliott et al., 2015; Lee et al., 2019).

Disulfiram is a second-line oral treatment for alcohol dependence (Bell and Smith, 1949), and has been used extensively in clinical settings and is generally well tolerated. The first clinical trial of disulfiram in PLWH on ART showed it was safe but resulted in no significant reactivation of latent virus (Spivak et al., 2014). However, in a subsequent dose escalation trial of disulfiram, administering 500 mg, 1000 mg or 2000 mg per day for 3 days, there was a clear dose dependent increase in cell associated unspliced HIV RNA (Elliott et al., 2015; Lee et al., 2019). Using pharmacokinetic and pharmacodynamic modelling of disulfiram and related metabolites, higher exposure predicted greater increases in cell associated unspliced but not plasma HIV RNA (Lee et al., 2019).

Unfortunately a subsequent trial evaluating high dose disulfiram (2000 mg per day for 28 days), together with intermittent short course vorinostat (a well tolerated histone deacetylase inhibitor (HDACi) and latency reversing agent), was terminated after the first two participants experienced unacceptable neurotoxicity (McMahon et al., 2022). Given the relative modest effects of disulfiram on latency reversal and the potential toxicities, this drug is no longer being pursued as an HIV cure intervention.

#### 3.2.2. PI3K

Activation of PI3Ks induces the phosphorylation of phosphatidylinositol 4,5-biphosphate (PIP2) into phosphatidylinositol 3,4,5-triphos- phate (PIP3) resulting in the downstream activation of AKT signalling (reviewed in (Manning and Toker, 2017)). Alkylphospholipids, a class of PI3K inhibitors, reduced HIV replication and induced cell death in HIV-infected macrophages (Chugh et al., 2008; Lucas et al., 2010). Similar to the effects on macrophages, another PI3K/AKT inhibitor, ripretinib, inhibited viral reactivation in both latently infected cell lines and cells from PLWH on ART (Cai et al., 2024). Inhibition of PI3K can also modulate HIV RNA splicing patterns, leading to a reduction of mature viral RNA, as well as a reduction in viral p24 production (Hillebrand et al., 2014). Taken together, PI3K inhibition could potentially play a role in latency promotion, although evaluation has not extended beyond investigations *in vitro*.

PI3K inhibition has also been shown to enhance latency reversal, when combined with a histone deacetylase inhibitor. Fimepinostat, a dual histone de-acetylase inhibitor (HDACi) and PI3K inhibitor, increased HIV latency reversal with lower cellular toxicity than the HDACi romidepsin (Gunst et al., 2019). However, it is unclear whether fimepinostat induced latency reversal required simultaneous inhibition of HDACs and PI3Ks, or if the effects were a result of HDAC inhibition alone. Further work on PI3K inhibitors as either latency promoting or reversing agents is warranted.

#### 3.2.3. mTOR

mTOR is a highly conserved threonine and serine kinase which impacts cellular signalling networks modulating homeostasis and metabolism (reviewed in (Panwar et al., 2023)). Rapamycin (also referred to as sirolimus) is an allosteric inhibitor of the mTOR1 domain, whilst more recently developed competitive ATP inhibitors, INK128 (sapanisertib (Heredia et al., 2015; Kadiyala et al., 2024) and MLN0128 (Caro-Vegas et al., 2019)), interfere with both mTOR complex 1 (mTORC1) and mTORC2 (reviewed in (Dowling et al., 2010)).

Rapamycin reduced constitutive expression of HIV RNA in transgenic mice containing *gag* and *pol* defective transgenes (Rai et al., 2013). This effect was potentially related to anti-inflammatory effects of rapamycin, including reduced release of inflammatory cytokines from CD4^+^ T cells (Martin et al., 2017) or reduced formation of tumour necrosis factor (TNF) containing inflammatory vesicles, as observed in cell line models of HIV infection following ionizing radiation to induce latency reversal (Pinto et al., 2020).

Rapamycin has also been evaluated *in vivo* in PLWH on ART in both observational and interventional studies and resulted in a decrease in the HIV reservoir. In an observational study of PLWH who underwent solid organ transplantation found that rapamycin use was associated with lower total HIV DNA levels (Stock et al., 2014). These findings were confirmed in a recent interventional clinical trial of rapamycin in otherwise healthy PLWH on ART, demonstrating a small but significant reduction in total HIV DNA, reduced CD4^+^ T cell proliferation and reduced PD-1 expression on CD8^+^ T cells, which is indicative of reduced immune exhaustion (Henrich et al., 2024).

Competitive ATP mTOR inhibitors are potentially more potent than rapamycin as they target both mTORC1 and mTORC2. The mTOR inhibitor INK128 inhibited *in vitro* replication of both CXCR4 and CCR5 tropic virus through inhibition of viral transcription, rather than viral entry (Heredia et al., 2015). INK128 mediated inhibition also interferes with viral Tat through preventing the phosphorylation of cyclin dependent kinase 9 (CDK9) (Besnard et al., 2016), blocking the formation of positive transcription elongation factor b (P-TEFb), an important host protein complex that interacts with Tat to achieve effective viral transcription (Zhu et al., 1997; Tahirov et al., 2010; Asamitsu et al., 2018). However, when INK128 was assessed *ex vivo* in 6 day culture with CD4^+^ T cells from PLWH on ART, there was no decline in intact proviral DNA (Kadiyala et al., 2024), suggesting that mTOR inhibition mediated reservoir decline *in vivo* is likely a consequence of complex indirect immune effects.

Broader tyrosine kinases inhibitors have been identified as mTOR inhibitors, including ponatinib, which inhibited reactivation of HIV in latently infected cell lines via reduction of AKT and mTOR phosphorylation (Huang et al., 2023); and ripretinib, which inhibited PI3K and mTOR, condensing the chromatin environment and inhibiting viral transcription (Cai et al., 2024).

More recently mTOR activators have been investigated as latency reversing agents. Two mTOR activators, the glycogen synthase kinase-3 inhibitors SB-216763 and tideglusib (the latter already in phase II clinical trials, NCT03692312) induced an increase in HIV RNA production in supernatant in *ex vivo* CD4^+^ T cells from PLWH via enhanced NF-κB signalling (Gramatica et al., 2021). However, when tideglusib was given to SIV-infected rhesus macaques, there was no evidence of latency reversal, which was likely the result of poor pharmacokine- tic/pharmacodynamic activity *in vivo* (Gramatica et al., 2021).

### 3.3. IL-15

The pro-inflammatory cytokine interleukin 15 (IL-15) is an important chemokine for the development of memory T cells responses (Richer et al., 2015). Signalling through the IL-15 pathway has effects on both HIV replication as well as HIV-specific immunity. IL-15 signalling has been shown to activate latent HIV, both increasing viral transcription (Jones et al., 2016) and viral p24 expression (Jones et al., 2016; Holmberg et al., 2024). Cotreatment with compounds that upregulate signal transducer and activator of transcription 5 (STAT5) signalling, including 3-hydroxy-1,2,3-benzotriazin-4(3H)-one (HODHBt (Copertino et al., 2023; Howard et al., 2024)) and isotretinoin (Howard et al., 2024) have enhanced IL-15 mediated latency reversal.

One possible limitation of IL-15 for HIV cure strategies is the risk of inducing proliferation of infected memory CD4^+^ T cells, a key reservoir for HIV (Chomont et al., 2009), leading to an expansion of the HIV reservoir (Manganaro et al., 2018). IL-15 enhanced the survival of unstimulated, and the expansion of stimulated, CCR5+ CD4^+^ T cells both in *in vitro* primary CD4^+^ T cells and *ex vivo* humanised mice, with a concurrent increase in viral p24 (Y. Li et al., 2023). IL-15 can also enhance CD4^+^ T cell permissiveness to infection, via an increase in sterile alpha motif domain and histidine-aspartic domain-containing protein 1 (SAMHD1) phosphorylation following Janus kinase (JAK) activation (Manganaro et al., 2018). Viral proteins have reciprocal effects on IL-15 expression, as demonstrated by viral protein Nef induced IL-15 release by monocytes and macrophages (Quaranta et al., 1999; Giordani et al., 2000), priming cells to be susceptible to infection.

In addition to direct effects on virus replication and proliferation of infected cells, IL-15 also has immune enhancing properties. In PBMCs from PLWH, the administration of IL-15 *ex vivo* enhanced the survival, activation and cytotoxicity of HIV specific CD8^+^ T cells (Mueller et al., 2003). When IL-15 was administered together with an HIV vaccine in rhesus macaques, there was an increase in central memory and effector memory CD8^+^ T cells (Li et al., 2010). Finally, IL-15 can also enhance natural killer (NK) cell responses against HIV. This was demonstrated in an *in vitro* infected CD4^+^ T cell model whereby cells treated with IL-15 and autologous NK cells resulted in a reduction in viral p24 expression and total HIV DNA (d’Ettorre et al., 2012). IL-15 also restored NK cell viability after HDACi treatment, though interestingly HDACi interfered with IL-15 induced p24 expression in an *in vitro* primary CD4^+^ T cell model of HIV infection (Covino et al., 2022).

The direct effects of IL-15 on the reservoir and its immune enhancing effects are best evaluated using *in vivo* models. The IL-15 superagonist N-803 (previously ALT-803 (Han et al., 2011) is a safe product now in clinical development. In ART-treated SIV-infected Mauritian cynomolgus macaques, N-803 induced expansion of SIV specific CD8^+^ T cells (Harwood et al., 2022). Similarly in ART-treated SHIV-infected rhesus macaques, N-803 increased SHIV specific CD8^+^ T cell and NK cell recruitment to B-cell follicles (Webb et al., 2020), thus enhancing immune cell penetration into a known sanctuary site for the HIV reservoir (reviewed in (Bronnimann et al., 2018)).

As a result of these promising pre-clinical data, N-803 has been evaluated in two phase I trials in PLWH. In both studies N-803 was shown to be safe, and when given alone increased the number of NK cells (Miller et al., 2022). In a second pilot study of six participants, N-803 was co-administered with a single infusion of haploidentical related donor NK cells and resulted in a decrease in both viral RNA and DNA in lymph nodes (Miller et al., 2024). N-803 is now being combined with other interventions such as broadly neutralizing antibodies (NCT05245292 and NCT04340596) in clinical trials that are currently enrolling participants.

### 3.4. Estrogen

Estrogen signalling can suppress HIV replication through several pathways. In relation to HIV transcription, 17β-oestradiol (17βE2) signalling increased Sp1 directed LTR mediated transcription in a latently infected cell line (Katagiri et al., 2006). However, 17βE2 also inhibited HIV transcription and expression of p24 via an estrogen receptor (ERα) complex with β-catenin that occluded the LTR in an *in vitro* infection model (Szotek et al., 2013).

Estrogen signalling can also interact with the viral protein Tat. Estrogen attenuated Tat induced IL-2 production and NF-κb signalling in human umbilical vein endothelial cells (Lee et al., 2004) while 17α-oestradiol (17αE2) protected against Tat induced neuronal damage (Datta et al., 2023). Furthermore, 17βE2 has been shown to inhibit Tat mediated viral transcription in an astrocyte cell line *in vitro* model (Heron et al., 2009).

In relation to HIV latency, estrogen receptor alpha (ERα) signalling inhibits T cell receptor (TCR) mediated proviral reactivation, whilst antagonism of ERα signalling enhanced reactivation by latency reversing agents (Das et al., 2018). As a consequence of this effect, CD4^+^ T cells from women compared to men living with HIV on ART were more sensitive to 17βE2 mediated inhibition of HIV transcription, but also showed greater HIV reactivation following ERα antagonism (Das et al., 2018). Consistent with the *in vitro* findings, in PLWH on ART, women compared to men had lower constitutive expression of cell associated multiply spliced HIV RNA (Scully et al., 2019) but also a slower decline in HIV DNA (Gianella et al., 2022).

As a strategy to enhance latency reversal, tamoxifen, a partial agonist of estrogen receptors, was evaluated in post-menopausal women with HIV on ART. In a prospective randomised clinical trial, tamoxifen together with the HDACi vorinostat, compared to vorinostat alone, led to no significant difference in changes in cell associated HIV RNA (Scully et al., 2022). Despite the disappointing findings in this clinical trial, the effects of estrogen on HIV transcription and the differences in the HIV reservoir between men and women warrant further investigation.

### 3.5. Circadian proteins

We and others have demonstrated that levels of HIV RNA in plasma (Zeichner et al., 1996) and in CD4^+^ T cells (Elliott et al., 2015; Stern et al., 2021) significantly vary with the time of day, leading to new insights that circadian proteins can impact HIV transcription. The transcription factors brain and muscle ARNT-like 1 (BMAL1, also known as ARNTL) protein and circadian locomotor output cycles kaput (CLOCK) protein form a heterodimer in the nucleus (Hogenesch et al., 1998) to enhance transcription at e-box elements in DNA (Gekakis et al., 1998). The HIV LTR also contains e-box elements (Ou et al., 1994) and we have shown that the BMAL1-CLOCK heterodimer enhances HIV transcription specifically via e-box 2 of the HIV LTR (Chang et al., 2018).

Similarly, the regulatory protein retinoic acid receptor-related orphan receptors (ROR) is another transcription factor family with circadian variation(reviewed in (Hastings et al., 2018)). ROR-α upre- gulates BMAL1 (Sato et al., 2004; Akashi and Takumi, 2005; Guillau-mond et al., 2005; Takeda et al., 2012), which in turn upregulates HIV transcription (Borrmann et al., 2023b). The analogue RORC2 can also enhance HIV transcription through direct binding to the LTR, with its inhibition by GSK261805A reducing p24 expression in *ex vivo* CD4^+^ T cells from PLWH (Salinas et al., 2021). RORs have been further targeted with inhibitors such as GSK2981278 and inverse agonists such as GSK805, which both reduced BMAL1 transcription, protein expression and viral activity (Borrmann et al., 2023b).

Circadian proteins can also suppress HIV replication and therefore could be exploited for block and lock strategies. The CLOCK-BMAL1 dimer upregulates a BMAL1 suppressing factor, REV-ERBα, which when activated with agonists, GSK2667 and SR9009, decreased LTR driven transcription in a latently infected cell line (Borrmann et al., 2020). Similarly, inhibition of salt inducible kinases (SIK), which negatively regulate circadian variation (Jagannath et al., 2013), inhibited LTR dependent protein expression, as shown with SIK inhibitors (HG-9-91-01, YKL-05-099, and ARN-3236) in a primary cell model of HIV infection (Borrmann et al., 2023a). This effect was hypothesised to be due to nuclear retention of the cyclic adenosine monophosphate (cAMP) response element-binding (CREB) protein regulated transcription coactivator 2 (CRTC2) interfering with RNA polymerase 2 interactions with the HIV LTR (Ma et al., 2021). The circadian regulatory system has many points where it can influence LTR driven transcription of HIV, and compounds which modulate these pathways will be of interest for both latency reversal and transcriptional silencing-based approaches.

### 3.6. ATF4

Activating transcription factor 4 (ATF4) is able to drive both direct LTR mediated transcription (Jiang et al., 2017; Lee et al., 2018), as well as enhance Tat mediated transcription (Caselli et al., 2012). ATF4 is also a key factor in the integrated stress response (reviewed in (Costa-Mattioli and Walter, 2020; Tian et al., 2021)), whereby its activation leads to the production of cytosine-cytosine-adenosine-adenosine-thymidine (CCAAT) enhancer-binding-protein (C/EBPs) homologous protein (CHOP, also known as GADD153) (Palam et al., 2011), which can lead to cell death (Zinszner et al., 1998). This mechanism may explain the observed decline of HIV DNA in *ex vivo* CD4^+^ T cells from PLWH after stimulation with the ATF4 agonist HA15 (D. Li et al., 2023). Interestingly, selenium administration also increased ATF4 signalling and reversed HIV latency in primary CD4^+^ T cells (Jiang et al., 2017). Inhibition of the forkhead box O1 (FOXO1) protein is another route by which ATF4 can be upregulated, as well as by inducing endoplasmic reticulum stress (Vallejo-Gracia et al., 2020), a potential strategy to achieve apoptosis of reactivated cells.

The strategies targeting host factors to modulate HIV transcription are summarised in Table 2, and relevant small molecule compound structures displayed in Supplementary Fig. 1B.

## 4. Host factors that alter survival of an infected cell

The “shock and kill” approach assumes that cells producing viral proteins will result in cell death via virus mediated cytopathy or immune mediated clearance. However, current latency reversal agents have shown that in the absence of additional interventions latently infected cells are resistant to cell death (Xing and Siliciano, 2013; Rasmussen et al., 2016; Zerbato et al., 2019). Several recent single-cell transcriptomic studies using *ex vivo* CD4^+^ T cells from PLWH on ART have revealed that latently infected cells have distinct transcriptomic features that favour proliferation, proviral silencing and survival (Collora et al., 2022; Clark et al., 2023; Sun et al., 2023, 2024; Wei et al., 2023; Wu et al., 2023). These studies represent an opportunity to identify new host targets to eliminate the HIV reservoir and are summarised in Table 3.

One single cell whole transcriptome analysis used a technique called focused interrogation of cells by nucleic acid detection and sequencing (FIND-seq) identified HIV DNA+ cells (HIV *gag* sequences) in memory CD4^+^ T cells from PLWH on ART who started ART during chronic infection. The authors showed that there were differentially expressed genes between HIV DNA+ and DNA-ve cells. HIV DNA+ cells exhibited pathways associated with prosurvival and proliferation (Clark et al., 2023).

In another study using another single-cell multi-omic sequencing called ECCITE-seq (expanded CRISPR-compatible cellular indexing of transcriptomes and epitopes by sequencing) on CD4^+^ T cells from six participants, prior to and following suppressive ART, there was an increase in prosurvival and protective factors such as B-cell lymphoma 2 protein (BCL-2) and Serpin family B Member 9 (SERPINB9) in HIV DNA+ RNA+ cells, making these cells resistant to immune cell mediated killing (Collora et al., 2022). This technique was limited to cells that expressed HIV RNA and thus provided no additional insights into factors favouring survival of transcriptionally silent cells.

DOGMA-seq (which enables the analysis of epigenetic modifications, transcriptomes and surface proteins) identified that in both HIV DNA+ and DNA+ RNA+ cells, there was increased gene expression of Ikaros family zinc finger 3 (IKZF3) and chromatin accessibility (Wei et al.,2023. ). IKZF3 is a transcription factor that induces cell proliferation, and co-expression of pro-survival and proliferation genes such as interleukin 21 (IL-21), baculoviral IAP repeat containing protein 5 (BIRC5) and the marker of proliferation Ki-67 (MKI67) which were detected in transcriptionally active cells, highlighting that these infected cells can adopt prosurvival and proliferative features (Wei et al., 2023).

ASAP-seq (ATAC [assay for transposase accessible chromatin] with select antigen profiling by sequencing), which allows surface and epigenetic marker analysis of cells containing viral DNA, demonstrated that in CD4^+^ T cells from PLWH on ART there was increased accessibility to genes that are involved in cell activation and proliferation including the AP-1 family (FOS and JUN) and BACH1 transcription factor motifs (Wu et al., 2023), indicating that reservoir cells are primed for reactivation and expansion.

PheP-seq (phenotypic and proviral sequencing) allows for the simultaneous determination HIV genome ‘intactness’ and detection of a range of cell surface markers using a single-cell barcoding approach. Here, cells harbouring intact genomes exhibited surface marker signatures consistent with increased resistance to immune-mediated killing (herpes virus entry mediator [HVEM], poliovirus receptor [PVR], programmed cell death ligand 1 [PDL-1]) and cell survival (CD44, CD28, CD127, IL-21R) (Sun et al., 2023).

Finally using PRIP-seq (parallel HIV-1 RNA integration site and proviral sequencing) analysis, which can determine the presence or absence of viral RNA as well as provide full length HIV DNA sequence and integration sites in CD4^+^ T cells from PLWH who are on and not on ART, it was shown that transcriptionally silent proviruses enriched in inaccessible chromatin sites (non-genic or satellite) during prolonged ART (Einkauf et al., 2022). However, it was also revealed that some clones of transcriptionally active intact proviruses, with integration sites proximal to activating epigenetic chromatin signals in their chromatin environment, could persist despite the selection pressure by cellular activation/proliferation which emphasises the need for pressure beyond activation to deplete the reservoir.

**Fig. 4. –:**
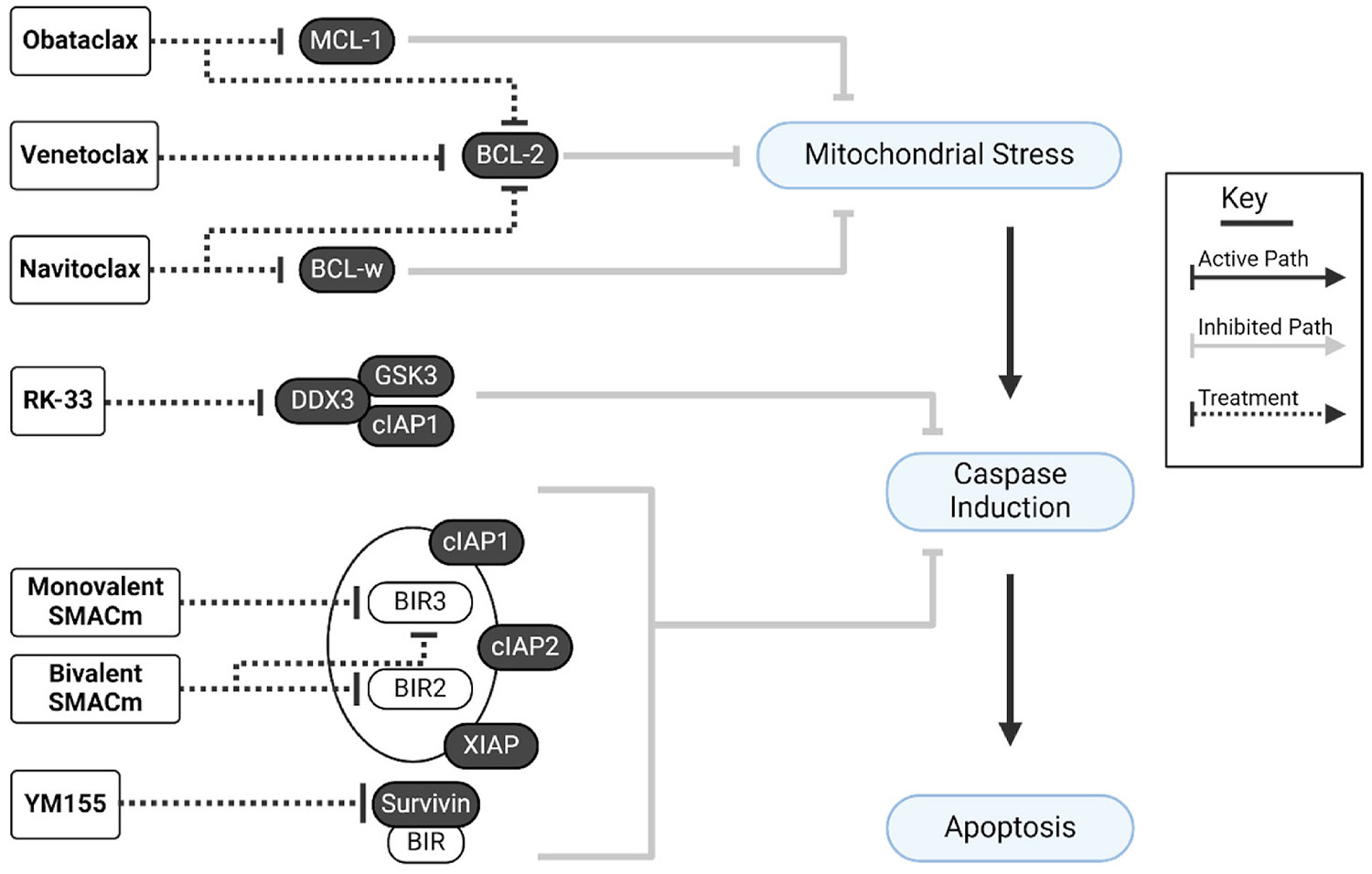
Targeting apoptosis for HIV reservoir clearance. Schematic of host factor targets which increase pro-apoptotic signalling pathways. Myeloid cell leukemia (MCL) and B Cell lymphoma (BCL) family proteins inhibit mitochondrial outer membrane permeabilization. Thus, their inhibition by obataclax, venetoclax and navitoclax respectively increases mitochondrial stress and downstream caspase induction. Inhibition of DEAD-box polypeptide 3 (DDX3) with RK-33 reduces DDX3-GSK2-cIAP1 complex mediated caspase suppression. Mimetics of the second mitochondrial derived activator of caspases (SMACm) and YM155 inhibit baculoviral inhibitor of apoptosis repeat (BIR) domains of several proteins, leading to increased caspase induction and downstream apoptosis. Created in BioRender. Shepherd, R. (2025) https://BioRender.com/4pg00b9.

These single cell sequencing studies have confirmed previous findings that prosurvival proteins are upregulated in HIV-infected cells, including proteins such as BCL-2 (Sharma et al., 2012; Cummins et al., 2017; French et al., 2020; Ren et al., 2020), survivin (Kuo et al., 2018) and cIAPs (Sharma et al., 2012; Campbell et al., 2018, 2020). Targeting these factors may indeed be a viable pathway for the selective elimination of the HIV reservoir (Fig. 4).

### 4.1. B cell lymphoma (BCL)-2

The B cell lymphoma 2 (BCL-2) family consists of multiple pro-survival proteins that are targeted by BH-3 mimetic drugs, such as venetoclax and navitoclax, which induce cell death via freeing BCL-2 associated X protein (BAX) and BCL-2 antagonist/killer protein (BAK) which leads to caspase activation and cell death (reviewed in (Diepstraten et al., 2022)). HIV infected cells that persist on ART over-express BCL-2 (Ren et al., 2020; Clark et al., 2023) and several groups have demonstrated that inhibition of BCL-2 with either venetoclax or obatoclax reduced infected cells *ex vivo* using cells from PLWH on ART and in an ART-treated HIV-infected humanised mouse model (Cummins et al., 2016, 2017; Arandjelovic et al., 2023; Zhou et al., 2023; Kadiyala et al., 2024). Our group recently demonstrated that after treatment of *ex vivo* CD4^+^ T cells from PLWH on ART with venetoclax, both total and intact HIV DNA significantly declined in a dose dependent manner (Arandjelovic et al., 2023). Furthermore, when venetoclax was administered to humanised mice on ART, either alone or combined with an inhibitor of the pro-survival protein myeloid cell leukemia protein (MCL1), S63845, there was a significant delay in viral rebound after cessation of ART (Chandrasekar et al., 2022; Arandjelovic et al., 2023). These assays demonstrate the potential ability of venetoclax to reduce the size of the reservoir.

Navitoclax (ABT263) is another BCL-2 inhibitor with minimal activity against BCL-w (also known as BCL2L2) (Tse et al., 2008). When PBMCs from PLWH on ART were treated with multiple cycles of a combination of latency reversal agents (ingenol-3,20-dibenzoate and JQ1), pro-apoptotic drugs (navitoclax and the autophagy inhibitor SAR405) and antivirals (the integrase inhibitor raltegravir and the attachment inhibitor BMS-626529) there was a depletion of cells that could produce virus *in vitro,* and *in vivo* using a mouse model (Li et al., 2020; M. Li et al., 2023). Another BCL-2 inhibitor that can also inhibit MCL1, obatoclax (Nguyen et al., 2007), reactivated latent virus in CD4^+^ T cells and PBMCs from PLWH on ART *ex vivo*, although induction of apoptosis was only shown in cell lines (Zhou et al., 2023). Another study using obatoclax observed selective reduction of intact HIV DNA in PBMCs from PLWH on ART *ex vivo* (Kadiyala et al., 2024). Taken together these studies demonstrate the important role that BH3 proteins play in survival of the HIV reservoir and that multiple agents that inhibit these proteins can reduce the HIV reservoir using a range of pre-clinical models.

Given the promising pre-clinical findings, a clinical trial of venetoclax in PLWH on ART is currently ongoing in Aarhus, Denmark and Melbourne, Australia. The trial will evaluate the maximum tolerated dose of venetoclax and will then administer three two-week cycles to determine if venetoclax can deplete the HIV reservoir *in vivo* (NCT05668026).

### 4.2. Survivin

BIRC5, also known as survivin, is the smallest protein in the inhibitors of apoptosis protein (IAP) family with one baculovirus IAP repeat (BIR) domain (reviewed in (Garg et al., 2016)). Survivin is upregulated by HIV viral protein R (Vpr) (Zhu et al., 2003). Survivin lacks multiple BIR domains that are present in cIAP1 (BIRC2), cIAP2 (BIRC3) and XIAP (BIRC4) ((Verdecia et al., 2000), reviewed in (Cetraro et al., 2022)). Other domains such as the caspase recruitment domains (CARD), ring finger domain (RING) and ubiquitin associated (UBA) domains are also absent in survivin (reviewed in (Garg et al., 2016)). Survivin inhibits apoptosis by interacting with other proteins such as pro-caspase 9 via hepatitis B X-interacting protein (HBXIP) (Marusawa et al., 2003), XIAP (Dohi et al., 2004), and SMAC (Song et al., 2003; Sun et al., 2005), to suppress caspase 9. Proteomic analysis of an *in vitro* model of HIV infection demonstrated upregulation of survivin and OX40 in productively and latently infected cells (Kuo et al., 2018). OX40, a member of the tumour necrosis factor receptor (TNFR) superfamily, controls survivin expression and is involved in memory T cell clonal expansion (Song et al., 2005). OX40 was also upregulated in *ex vivo* CD4^+^ T cells from PLWH on ART that harboured viral DNA and was enriched in expanded HIV-1 clones, potentially playing a role in persistence (Kuo et al., 2018).

YM155 is a small molecule inhibitor of survivin and has been investigated in a clinical trial as a therapeutic for breast cancer (Clemens et al., 2015). *In vitro*, YM155 treatment reduced the frequency of HIV+ CD4^+^ T cells. *Ex vivo,* in PBMCs from PLWH on ART, YM155 cultured for 7 days in the absence of CD8^+^ T and NK cells reduced total and intact HIV DNA as well as reduced clonally expanded sequences (Kuo et al., 2018). Therefore, survivin may also represent an attractive target to reduce the HIV reservoir.

### 4.3. DDX3

DDX3 (DEAD-box polypeptide 3) is an ATP-dependent RNA helicase that regulates RNA metabolism (reviewed in (Bol et al., 2015)) and is crucial for HIV RNA export and translation (reviewed in (Stunnenberg et al., 2018)). DDX3 is also known to regulate apoptosis by interacting with death receptors such as TRAIL-R2 (TNFR superfamily member 10b) and TNFR1. It forms protein complexes with other antiapoptotic proteins such as cIAP1, and glycogen synthase kinase 3 (GSK3), to antagonise apoptosis signalling (Bol et al., 2015). DDX3 expression is increased in various cancers and therefore a target for novel therapeutics (reviewed in (Mo et al., 2021)).

RK-33 is a preclinical small molecule inhibitor that binds to the ATPase domain of DDX3 (Bol et al., 2015). In the latently infected cell line, J-Lat 11.1 and a primary cell model of HIV latency, RK-33 increased HIV transcription consistent with reversing HIV latency and increased cell death in transcriptionally active infected cells (Rao et al., 2021). Inhibition of RNA helicase activity using RK-33 in uninfected primary CD4^+^ T cells, compared to non-treated cells, showed differential gene expression with down regulation of two host factors known to regulate apoptosis, survivin and HSP70 (Rao et al., 2021). *Ex vivo* CD4^+^ T cells from PLWH treated with DDX3 inhibitors, RK-33 or FH1321, showed latency reversal and death in transcriptionally active cells, identified by the loss of intracellular HIV RNA using FISH-Flow (Rao et al., 2021). FISH-flow uses immunohistochemistry and RNA probes that are specific to HIV gag, so these cells have not necessarily overcome all the transcriptional blocks to enable splicing (Rao et al., 2021). The combination of the SMACm AZD5582 and DDX3 inhibition using the compound FH1321 led to depletion of the inducible HIV reservoir in *ex vivo* PBMCs from PLWH (Jansen et al., 2024). The approaches to altering survival of infected cells are summarised in Table 4 and relevant chemical structures in Supplementary Fig. 1C.

## 5. Enhance immune-mediated control

Successful strategies for durable control of HIV will likely require a combination of different treatments. One important component of combinations will be strategies to enhance the host’s anti-HIV immune response to both clear infected cells but also maintain long term control for any low-level virus that may persist.

### 5.1. Immune checkpoints

Immune checkpoint molecules, such as programmed cell death 1 (PD-1) and cytotoxic T lymphocyte associated protein 4 (CTLA-4), are surface proteins upregulated on cells following chronic antigen stimulation, including in the setting of HIV infection (Day et al., 2006; Petrovas et al., 2006; Trautmann et al., 2006; Kaufmann et al., 2007). Upregulation of immune checkpoint molecules dampens anti-HIV immune responses and contributes to viral persistence, by restricting reactivation and maintaining HIV latency (Khoury et al., 2017; Evans et al., 2018; Fromentin et al., 2019). Monoclonal antibody therapeutics that block the checkpoint inhibitors PD-1 (and its ligand, PD-L1), CTLA-4, and lymphocyte-activation gene 3 (LAG-3) are currently licensed for treatment of multiple cancers, where they function to enhance anti-cancer T cell responses (Shiravand et al., 2022).

In the setting of HIV infection, *ex vivo* blockade of checkpoint inhibitors, such as PD-1, enhanced the proliferation and functionality of HIV-specific T cells (Trautmann et al., 2006; Grabmeier-Pfistershammer et al., 2017; Chiu et al., 2022) and reversed HIV latency (Van der Sluis et al., 2020a). PD-1 blockade reduced viremia in SIV-infected non-human primates not on ART (Velu et al., 2009, 2022; Mylvaganam et al., 2018), however there was minimal effect of anti-PD1 when administered during ART suppression (Bekerman et al., 2019). These successful studies in animal models have inspired multiple case studies and clinical trials in PLWH (reviewed in (King and Lewin, 2024)). Most of these studies have been undertaken in PLWH with cancer, and demonstrated an increase in HIV-specific T cell responses (Lau et al., 2021; Lavole et al., 2021) and decreased HIV reservoir size (Evans et al., 2018; Fromentin et al., 2019; Uldrick et al., 2022) in some but not all participants. A number of ongoing clinical trials aim to better understand the safety and antiviral effects of checkpoint blockade in PLWH without cancer ((King and Lewin, 2024), NCT05187429).

### 5.2. Toll-like receptors

Toll-like receptors (TLRs) are pattern recognition receptors that recognise microbial membrane components or viral or bacterial DNA. TLRs signal through the MYD88 (myeloid differentiation primary response gene 88) or TRIF (TIR [toll/interkukin1 receptor] domain containing adapter inducing interferon-β) pathways, leading to expression of inflammatory cytokines and other immune activating proteins (Kawasaki and Kawai, 2014). TLR agonists are being investigated as immunostimulatory agents to either reverse HIV latency by reactivating resting T cells, or to enhance antiviral immune responses. *In vitro*, agonists of TLRs 2,3,4,5,7,8 and 9 reactivated HIV latency (Equils et al., 2001; Scheller et al., 2004; Schlaepfer et al., 2006; Thibault et al., 2009; Schlaepfer and Speck, 2011; Nowak et al., 2012; Novis et al., 2013; Offersen et al., 2016; Alvarez-Carbonell et al., 2017; Larson et al., 2017; Rochat et al., 2017; Macedo et al., 2018; Ganor et al., 2019; Meås et al., 2020); however, *in vivo* in both animal models and human clinical trials of either TLR7 or TLR9 agonists, there was limited evidence of latency reversal (Vibholm et al., 2019; Riddler et al., 2021; SenGupta et al., 2021; Gunst et al., 2023).

TLR9 agonists have advanced the furthest in clinical trials for HIV cure, where results have been mixed. The TLR9 agonist, lefitolimod, activates plasmacytoid dendritic cells, CD8^+^ T cells and NK cells, and in a non-randomised single arm study led to an increase in plasma HIV RNA in virally suppressed PLWH (Vibholm et al., 2017). However, when lefitolimod was administered to PLWH at the time of an analytical treatment interruption (ATI), either alone (Vibholm et al., 2019) or in combination with broadly neutralizing antibodies (3BNC117 and 10–1074) (Gunst et al., 2023), there was no increase in plasma RNA nor improvements in viral control following an ATI.

The TLR3 agonist Poly I:C has also been investigated in combination with a therapeutic vaccine. This combination reduced levels of HIV DNA in lymphoid tissue and delayed viral rebound following cessation of ART in humanised mice (Cheng et al., 2018); however, in ART-suppressed PLWH, Poly I:C had no effect on the HIV reservoir (Saxena et al., 2019).

Another target being investigated for HIV cure is TLR7. In rhesus macaques, the TLR7 agonists GS-986 and GS-9620 caused intermittent increases in plasma HIV RNA in some (Lim et al., 2018), but not all (Del Prete et al., 2019) studies. Promisingly, combining a TLR7 agonist with a broadly neutralizing antibody (bNAb) in SHIV-infected rhesus macaques induced viral control following cessation of ART in ~50 % of animals in three separate studies (Borducchi et al., 2018; Moldt et al., 2022; Walker-Sperling et al., 2022), and a delay in viral rebound following cessation of ART in another (Hsu et al., 2021). However, control of virus replication off ART was not achieved if the TLR7 agonist was combined with a therapeutic adenovirus vectored vaccine (Borducchi et al., 2016; Walker-Sperling et al., 2022). TLR7 agonists have also progressed into clinical trials in PLWH. In one study, the TLR7 agonist vesatolimod did not impact HIV plasma RNA in individuals on ART (Riddler et al., 2021), while in another study it induced a modest delay in time to rebound following cessation of ART (SenGupta et al., 2021). Further understanding of the differences in efficacy of TLR7 agonists in animal models and human clinical trials will be important to design future effective clinical trials.

### 5.3. Cereblon

Cereblon is an E3 ubiquitin ligase which can be stimulated by the immunomodulatory imide drug (IMiD) pomalidomide, which is licensed for the treatment of relapsed/refractory multiple myeloma (rrMM), and AIDS-related Kaposi Sarcoma (KS)(US FDA, 2020). The immune-enhancing effects of pomalidomide are induced by binding to cereblon, which leads to the degradation of transcription factors Ikaros and Aiolos, subsequently increasing T cell expression of interleukin-2 (IL-2) (Gandhi et al., 2014). Multiple *in vitro* studies showed that pomalidomide had a stimulatory effect on T cells, NK cells and antibody-dependent cellular cytotoxicity (ADCC) (Hayashi et al., 2005; Reddy et al., 2008; Richardson et al., 2013) as well as inhibited the suppressive function of regulatory T cells (Galustian et al., 2009).

*Ex vivo* studies conducted on samples from PLWH on ART showed that pomalidomide treatment enhanced anti-HIV immunity. Pomalidomide was associated with a greater expansion of HIV-specific CD8^+^ T cells and increased HIV-specific cytotoxicity (Pascoe et al., 2023). Furthermore, treatment reduced NK cell dysfunction and improved NK cell polyfunctionality (Pascoe et al., 2022).

To date, pomalidomide has only been administered to PLWH as a treatment for concurrent cancer (Polizzotto et al., 2016; Polizzotto et al., 2019; (US FDA, 2020). Consistent with preclinical findings, pomalidomide increased T cell activation in PLWH treated for Kaposi Sarcoma, but there was no clear correlation between the immunophenotypic changes and treatment response (Lurain et al., 2023). A phase I/IIb clinical trial of pomalidomide in otherwise healthy PLWH on ART is currently underway in Aarhus, Denmark and Melbourne, Australia (NCT06660498).

### 5.4. Interferon-stimulated genes

Interferons (IFNs) promote the production of antiviral proteins through increased transcription of interferon-stimulated genes (ISGs) (Sugawara et al., 2019). Several studies suggest that HIV might evade immune detection by reducing the level and/or function of ISG proteins (Zhao et al., 2021). Exogenous administration of IFNs and the subsequent increase in proteins that can restrict HIV replication has therefore been suggested as a potential cure intervention (Pillai et al., 2012).

In preclinical studies, IFN treatment enhanced anti-HIV immunity (Lapenta et al., 2003) and inhibited HIV replication (Harman et al., 2011; Nasr et al., 2012) as well as the establishment of latency (Van der Sluis et al., 2020b). There have been multiple clinical trials in PLWH have shown that IFN therapy showing a vast range of immune-mediated effects including increased T cell activation (Manion et al., 2012), increased NK cell cytotoxicity (Papasavvas et al., 2019), increased GALT immune activation (Papasavvas et al., 2021) and in some settings, control of viral replication (Lane et al., 1988; Asmuth et al., 2010; Pillai et al., 2012; Azzoni et al., 2013). Furthermore, IFN treatment and the upregulation of ISGs that can restrict HIV replication correlated with declines in plasma HIV RNA (Abdel-Mohsen et al., 2014) and viremic control following cessation of ART, as long as IFNs were continued (Azzoni et al., 2013).

Recently, IFNs were tested in combination with other therapies aimed at achieving an HIV cure. The combination of IFNs and broadly neutralizing antibodies was well tolerated and resulted in sustained viral suppression in the majority of participants during a 26-week ATI (Tebas et al., 2023). However, IFN therapy has multiple adverse effects which may limit the ability to progress IFN as part of curative strategies(Gara and Ghany, 2013).

### 5.5. Cytokines (IL-2, IL-7, IL-10)

Interleukins (ILs) are a group of cytokines that help modulate immune responses. *In vitro* studies on IL-2, IL-7 and IL-15 initially established their ability to prime latently infected cells for recognition by CD8^+^ T cells (Jones et al., 2016). Subsequent studies on IL-2 and IL-7 showed mixed results with modest effects, if any, on the reservoir or viral control (reviewed in (Pett et al., 2010)).

IL-10 dampens the immune response by reducing antigen-expression and triggering T cell anergy (Hoves et al., 2006; Wilson and Brooks, 2011; Mittal et al., 2015). In SIV-infected rhesus macaques on ART, plasma levels of IL-10 correlated with survival of T cells harbouring SIV DNA (Harper et al., 2022), whereas blocking IL-10 and PD-1 was associated with SIV-control following cessation of ART (Pereira Ribeiro et al., 2024). Collectively, these data suggest that targeting IL-10 signalling may improve anti-HIV immunity and limit viral persistence in PLWH.

**Fig. 5. –:**
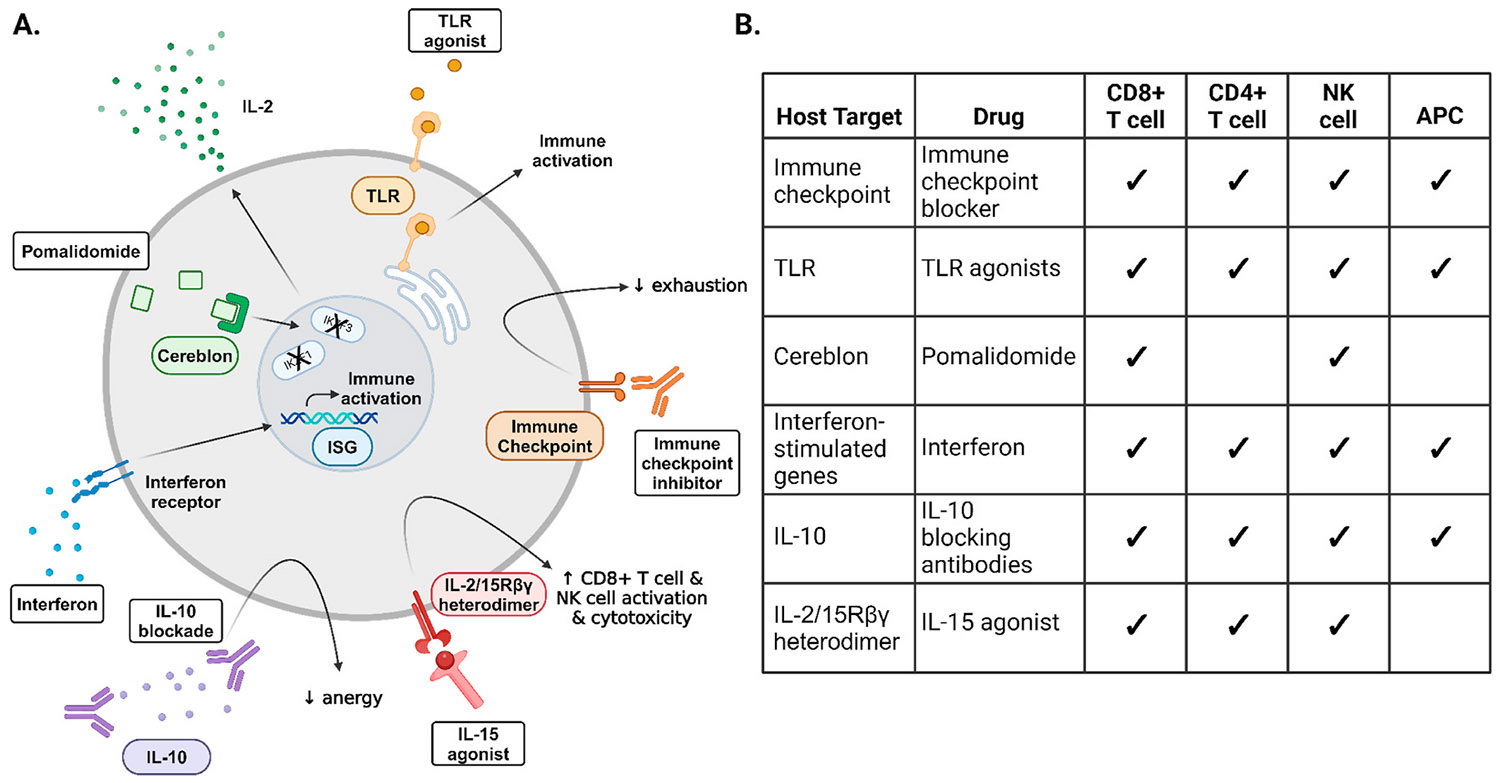
Immune mediated control for clearing the HIV reservoir. (A) Schematic of drugs used to target host immune pathways, including: immune checkpoint inhibitors; toll like receptor (TLR) agonists; cereblon which degrades IKAROS family zinc finger protein 1 (IKZF1) and 3 (IKZF3); interferon stimulated genes (ISG); interleukin 10 (IL-10); and interleukin 2/15 receptor beta gamma (IL-2/15Rβγ). (B) Cellular distribution of different host immune targets, including CD8^+^ T cells, CD4^+^ T cells, natural killer cells (NKs) and antigen presenting cells (APCs). Ticks indicate expression of target in the indicated cell type. Created in BioRender. King, H. (2025) https://BioRender.com/h07m237.

Promising host targeting strategies for immune enhancement discussed above are summarised in Table 5, Fig. 5 and relevant small molecule compounds displayed in Supplementary Fig. 1D.

## 6. Summary and conclusion

Although current antiviral treatment almost exclusively targets viral proteins or enzymes with high selectivity, a large number of cure strategies target host proteins to either reduce the size of the reservoir or enhance HIV-specific immunity. A major challenge of targeting a host protein is the lack of specificity for HIV and therefore increased risk of adverse events. However, cure strategies are designed to be time limited, as opposed to ART which is lifelong. Therefore, it is possible that host-targeted therapy even if associated with adverse effects may be acceptable, providing efficacy is high and the adverse effects not severe and reversible. The acceptability of some toxicity for cure interventions was recently demonstrated in discussions with key stakeholders including civil society, in work done on a target product profile for cure (Lewin et al., 2021, 2025). Despite the significant progress in understanding the HIV reservoir, cure strategies tested in animal models and early phase experimental clinical trials, there remains a high need for fundamental discovery science to identify new targets and better understand current host targets and the impact of inhibiting or activating these pathways either alone or in combination.

## Supplementary Material

1

## Figures and Tables

**Table 1:** Host factors that are targeted for modulation of HIV infection.

Modulation of HIV infection					
Target	Effect on target	Intervention	Pre-clinical	Clinical trials	Reference
CCR5	Inhibition	Gene therapy - CRISPR, TALENs, ZFN	Humanised Mice (CRISPR) *In vitro* models	Yes (ZFN)	(Tebas et al., 2014, 2021; Xu et al., 2017; Romito et al., 2021; Schwarze et al., 2021)
	Inhibition	Maraviroc Rapamycin	Non-human primates (rapamycin) *In vitro* models	Yes	(Nicoletti et al., 2009; López-Huertas et al., 2017, 2020; Madrid-Elena et al., 2018; Lopez-Huertas et al., 2020; Varco-Merth et al., 2022)
	Inhibition	Antibody - leronimab	Non-human primates *In vitro* models	Yes	(Dhody et al., 2018; Chang et al., 2021a, 2021b, 2022)
CCR5/CXCR4	Inhibition	CCR5 CRISPR/Cas9 gene-therapy; CXCR4 fusion inhibitor C46	*In vitro* models	No	Khamaikawin et al. (2024)

CCR5 = C-C motif chemokine receptor 5; CXCR4 = C-X-C motif chemokine receptor 4; CRISPR = clustered regularly interspaced short palindromic repeats; Cas9 = CRISPR associated protein9; TALENs = transcription activator like effector nuclease; ZFN = zinc finger nuclease.

**Table 2:** Host factors that are targeted for modulation of HIV transcription.

Modulation of HIV transcription					
Target	Effect on target	Intervention	Pre-clinical	Clinical trials	Reference
ATF4	Activation	Amino Acid Deprivation Exogenous selenium HA15	*In vitro* models	No	(Jiang et al., 2017; D. Li et al., 2023)
BIRC2/BIRC3	Inhibition	SMACm	Humanised mice Rhesus macaques	No	(Bobardt et al., 2019; Nixon et al., 2020; Dashti et al., 2023; Jain et al., 2024,
BMAL1/RORC	Inhibition	GSK2981278 GSK805 GSK261805A	*In vitro* models	No	(Salinas et al., 2021; Borrmann et al., 2023b)
Estrogen	Activation	β-estradiol (E2)	*In vitro* models	No	Das et al. (2018)
	Inhibition	Fulvestrant	*In vitro* models	No	Das et al. (2018)
	Partial activation	Tamoxifen	*In vitro* models	Yes	Scully et al. (2022)
IL-15	Activation	N-803 superagonist Exogenous IL-15	*In vitro* models Humanised mice Non-human primates	Yes	(Dashti et al., 2023; Jones et al., 2016; Webb et al., 2020; Harwood et al., 2022; Y. Li et al., 2023; Miller et al., 2024)
mTOR	Inhibition	Rapamycin INK128 Ponatinib	*In vitro* models Humanised mice (INK128, rapamycin)	Yes (rapamycin)	(Heredia et al., 2015; Caro-Vegas et al., 2019; Huang et al., 2023; Kadiyala et al., 2024)
PI3K	Inhibition	Alkylphospholipids LY294002 Fimepinostat	*In vitro* models	No	(Chugh et al., 2008; Lucas et al., 2010; Hillebrand et al., 2014; Gunst et al., 2019)
PI3K/mTOR	Inhibition	Ripretinib	*In vitro* models	No	Cai et al. (2024)
	Activation	Tideglusib SB-216763	*In vitro models*	No	Gramatica et al. (2021)
PTEN	Inhibition	Disulfiram	*In vitro* models	Yes	(Spivak et al., 2014; Elliott et al., 2015; Lee et al., 2019; McMahon et al., 2022)
REV-ERB	Inhibition	GSK2667 SR9009	*In vitro* models	No	Borrmann et al. (2020)
SIK	Inhibition	HG-9-91-01 YKL-05-099 ARN-3236	*In vitro* models	No	Borrmann et al. (2023a)

ATF4 = activator of transcription factor 4; BIRC2 = baculoviral inhibitor of apoptosis repeat containing 2; BIRC3 = baculoviral inhibitor of apoptosis repeat containing 3; BMAL1 = brain and muscle arnt-like 1; RORC = retinoic acid receptor-related orphan receptor C; IL-15 = interleukin 15; mTOR = mammalian target of rapamycin; PI3K = phosphoinositide 3-kinase; PTEN = phosphatase and tensin homolog; REV-ERB = nuclear receptor D1; SIK = salt inducible kinase; SMACm = mimetics of the second mitochondrial derived activator of caspases.

**Table 3:** Single Cell Sequencing approaches that have identified new host targets in latently infected cells that are relevant for HIV cure strategies.

Single cell technique	Analysis	HIV target	Off-ART participants	On-ART participants	Host targets identified	Reference
ASAP-seq	Epigenetic Surface protein	HIV DNA+	6	4 (3 longitudinal)	Activation/proliferation AP-1 family (FOS and JUN), BACH1/2 motifs↑ accessibility in HIV DNA + cells (LN from unsuppressed and peripheral from ART suppressed)	Wu et al. (2023)
DOGMA-seq	Epigenetic Host Transcriptome HIV RNA Surface protein	HIV DNA+ and/or HIV RNA+	6	6 (longitudinal)	Genes in Cytotoxic T cell differentiation (GZMA, CTSC), T cell migration (ITGA4, ITGB7), Type I IFN responses (MX1, STAT1, STAT2), T cell differentiation transcription factors (FOSL2, REL, MAF, STAT5B), Cell survival gene (CFLAR – encoding c-FLIP that prevents T cell death) ↑ in HIV RNA + cells Co-expression of genes involved in cell division and differentiation (IKZF3 with IL-21, BIRC5, MKI67) distinguished HIV DNA + RNA− and RNA + vs uninfected cells	Wei et al. (2023)
ECCITE-seq	Host transcriptome HIV RNA TCR sequence Surface protein	HIV RNA+ (Only transcriptionally active reservoirs)	6	6 (longitudinal)	BCL-2↑ SERPINB9↑ in transcriptionally active reservoirs Persistent and large HIV RNA + cell clones that are enriched in GZMB + cytotoxic effector memory Th1 cells	Collora et al. (2022)
FIND-seq	Host transcriptome HIV RNA	HIV DNA+	–	5	Non-permissive profile for HIV transcription Genes in apoptosis inhibition (USP19 and LRRFIP2) and antigen-driven T cell proliferation (TLN1) ↑	Clark et al. (2023)
PheP-seq	Epigenetic Surface Protein	HIV DNA+	–	5 (2 longitudinal)	Upregulation of markers associated with cell survival including CD44, CD28, CD127, IL-21R Elevated levels of immune checkpoint markers including PD-1, TIGIT	Sun et al. (2023)

FIND-seq = focused interrogation of cells by nucleic acid detection and sequencing; USP19 = ubiquitin specific peptidase 19; LRRFIP2 = leucine rich repeat binding FLII interacting protein 2; TLN1 = talin 1; ECCITE-seq = expanded CRISPR-compatible cellular indexing of transcriptomes and epitopes by sequencing; TCR = T cell receptor; BCL-2 = B cell lymphoma 2; SERPINB9 = serine proteinase inhibitor B9; GZMB = granzyme B; Th1 = type 1 T helper cell; GZMA = granzyme A; CTSC = cathepsin C; ITGA4 = integrin A4; ITGB7 = integrin B7; IFN = interferon; MX1 = myxovirus dynamin like GTPase 1; STAT1 = signal transducer and activator of transcription 1; STAT2 = signal transducer and activator of transcription 2; FOSL2 = FOS like 2; REL = reticuloendotheliosis viral oncogene homolog, NF-kB subunit; MAF = musculoaponeurotic fibrosarcoma bZIP transcription factor; STAT5B = signal transducer and activator of transcription 5B; CFLAR = caspase 8 and fas associated via death domain (FADD) like apoptosis regulator = c-FLIP; IKZF3 = IKAROS family zinc finger 3; IL-21 = interleukin 21; BIRC5 = baculoviral inhibitor of apoptosis repeat containing 5; MKI67 = maker of proliferation Ki-67; ASAP-seq = ATAC with select antigen profiling by sequencing; FOS = FBJ murine osteosarcoma proto-oncogene, AP-1 transcription factor subunit; JUN = Jun proto-oncogene, AP-1 transcription factor subunit; BACH1/2 = BTB domain and CNC homolog 1/2; LN = lymph node; ART = antiretroviral therapy; PheP-seq = phenotypic and proviral sequencing; IL-21R = interleukin 21 receptor; TIGIT = T cell immunoreceptor with Ig and ITIM domains.

**Table 4:** Host factors that are targeted to reduce survival of the HIV reservoir.

Targeting survival of infected cells					
Target	Effect on target	Intervention	Pre-clinical	Clinical trials	Reference
BCL2	Inhibition	Venetoclax Navitoclax	*In vitro* models Humanised mice	Yes – recruiting	(Cummins et al., 2016; Cummins et al., 2017; Li et al., 2020; Chandrasekar et al., 2022; M. Li et al., 2023) (NCT05668026)
BCL2/MCL1	Inhibition	Venetoclax + S63845 Obatoclax	*In vitro* models Humanised Mice	No	(Arandjelovic et al., 2023; Zhou et al., 2023; Kadiyala et al., 2024)
BIRC5	Inhibition	YM155	*In vitro* models	No	Kuo et al. (2018)
DDX3	Inhibition	FH1321+ AZD5582 RK-33 + FH1321	*In vitro* models	No	(Jansen et al., 2024; Rao et al., 2021)

BCL-2 = B cell lymphoma 2; MCL1 = myeloid cell leukemia 1; BIRC5 = baculoviral inhibitor of apoptosis repeat containing 5 (survivin); DDX3 = DEAD-box polypeptide 3.

**Table 5:** Host factors that are targeted to enhance immune-mediated control of HIV.

Enhancing immune-mediated control					
Target	Effect on target	Intervention	Pre-clinical	Clinical trials	Reference
Cereblon	Activation	Pomalidomide	*In vitro* models	Yes (recruiting)	(Polizzotto et al., 2016, 2019; Pascoe et al., 2022, 2023; Lurain et al., 2023) (NCT06660498)
Cytokine Signalling	Activation	Exogenous IL-2	*In vitro* models	Yes	(Chun et al., 1999b; Dybul et al., 2002; Stellbrink et al., 2002; Abrams et al., 2009; Jones et al., 2016)
	Activation	Exogenous IL-7	*In vitro* models	Yes	(Levy et al., 2009; Sereti et al., 2009; Imamichi et al., 2011; Vandergeeten et al., 2013; Jones et al., 2016)
	Activation	Exogenous IL-15 + Isotretinoin	*Ex vivo* models	No	Howard et al. (2024)
	Inhibition	Antibody - Anti-IL-10	*In vitro* models Rhesus macaques	No	Harper et al., 2022; Pereira Ribeiro et al., 2024)
Immune checkpoints (i.e. PD-1, PD-L1, CTLA-4)	Inhibition	Antibodies Anti-PD-1 – i.e. Pembrolizumab Nivolumab; Anti-PDL-1 – i.e. Avelumab; Anti-CTLA-4 – i.e. Ipilimumab	*In vitro* models Rhesus macaques	Yes	(Trautmann et al., 2006; Velu et al., 2009, 2022;Grabmeier-Pfistershammer et al., 2017; Evans et al., 2018; Mylvaganam et al., 2018; Fromentin et al., 2019,; Van der Sluis et al., 2020a; Lau et al., 2021; Lavole et al., 2021; Chiu et al., 2022; Uldrick et al., 2022)
Interferon Stimulated Genes	Activation	Exogenous Interferons	*In vitro* models	Yes	(Lane et al., 1988; Lapenta et al., 2003; Asmuth et al., 2010; Harman et al., 2011,; Manion et al., 2012; Nasr et al., 2012; Pillai et al., 2012; Azzoni et al., 2013; Abdel-Mohsen et al., 2014; Papasavvas et al., 2019, 2021; Van der Sluis et al., 2020b; Tebas et al., 2023)
TLR7	Activation	GS-986 GS-9620 Vesatolimod	*In vitro* models Rhesus macaques	Yes	(Borducchi et al., 2016, 2018; Lim et al., 2018; Del Prete et al., 2019; Hsu et al., 2021; Riddler et al., 2021; SenGupta et al., 2021; Moldt et al., 2022; Walker-Sperling et al., 2022)
TLR9	Activation	Lefitolimod, CpG-ODN (*in vitro*)	*In vitro* models	Yes	(Scheller et al., 2004; Nowak et al., 2012; Offersen et al., 2016; Vibholm et al., 2017,, 2019; Gunst et al., 2023)

PD-1 = programmed cell death 1; PDL-1 = programmed cell death ligand 1; CTLA-4 = cytotoxic T-lymphocyte associated protein 4; TLR7 = toll like receptor 7; TLR9 = toll like receptor 9; IL-2 = interleukin 2; IL-7 = interleukin 7; IL-15 = interleukin 15; IL-10 = interleukin 10.

## Data Availability

No data was used for the research described in the article.

## Appendix A. Supplementary data

Supplementary data to this article can be found online at https://doi.org/10.1016/j.antiviral.2025.106216.
