# Supplementary material for: Host-directed approaches in the pursuit of a cure for HIV
Source: Antiviral Res. Author manuscript; Available in PMC 2026 May 14. (PMC13171291; doi:10.1016/j.antiviral.2025.106216)
Supplement: 1 [file NIHMS2168606-supplement-1.docx]

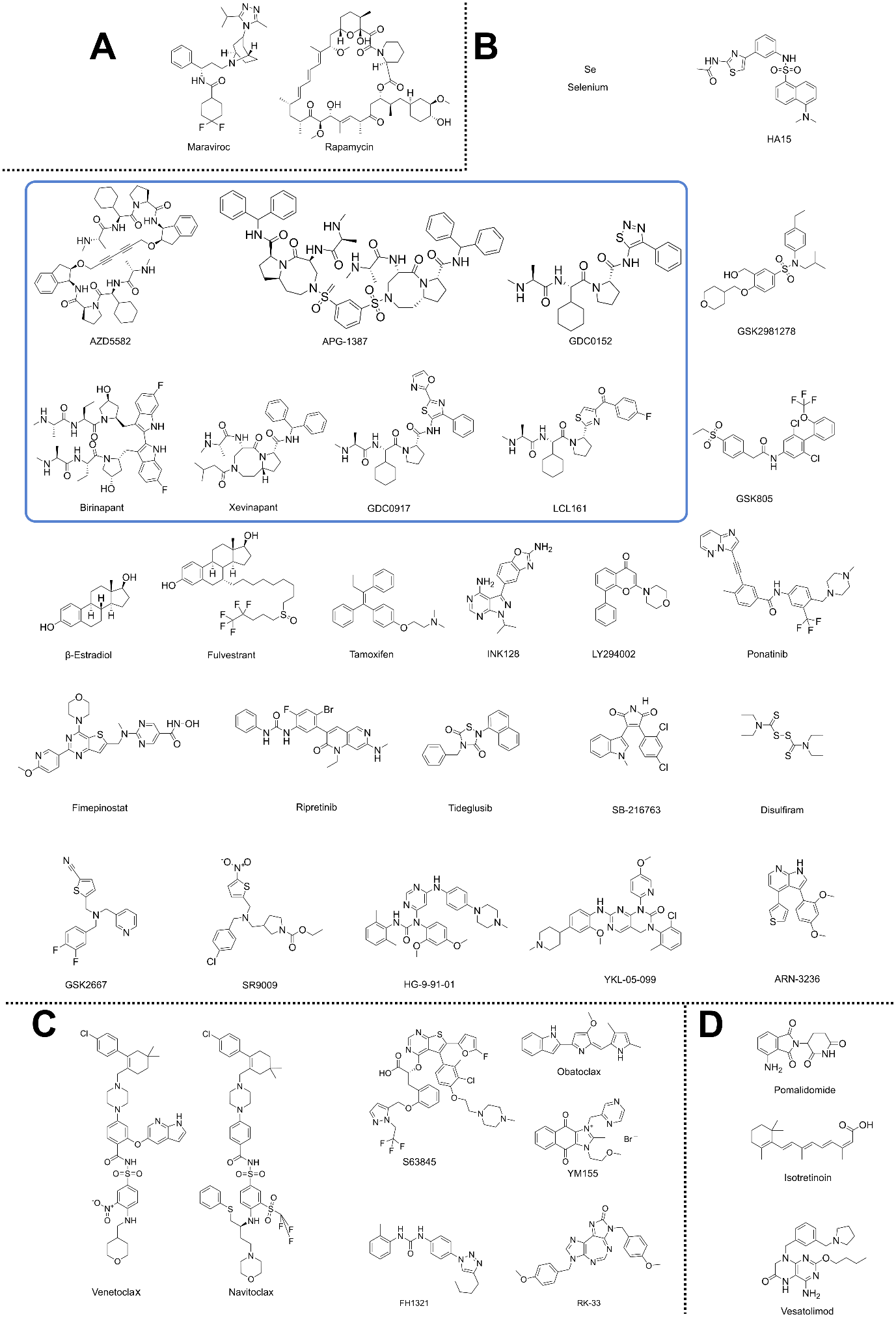
Supplementary Figure 1 – Chemical structure of host targeting small molecule compounds. This figure contains the structure of host targeting small molecule compounds which are publicly available. (A) Compounds that modulate HIV infection. (B) Compounds that modulate HIV transcription. Surrounded by blue are mimetics of the second mitochondrial derived activators of caspases (SMACm). (C) Compounds that alter the survival of infected cells. (D) Compounds that enhance immune mediated control of HIV. Compounds are included relative to the section on the review in which they were first mentioned.
